# Association Between Antibiotic Treatment and the Efficacy of Intravesical BCG Therapy in Patients With High-Risk Non-Muscle Invasive Bladder Cancer

**DOI:** 10.3389/fonc.2021.570077

**Published:** 2021-04-02

**Authors:** Sahyun Pak, Sun-Young Kim, Sung Han Kim, Jae Young Joung, Weon Seo Park, Jinsoo Chung, Kang Hyun Lee, Ho Kyung Seo

**Affiliations:** ^1^ Department of Urology, Center for Urologic Cancer, National Cancer Center, Goyang, South Korea; ^2^ Department of Urology, Hallym University Kangnam Sacred Heart Hospital, Hallym University College of Medicine, Seoul, South Korea; ^3^ Biometrics Research Branch, Research Institute, National Cancer Center, Goyang, South Korea; ^4^ Division of Tumor Immunology, Research Institute, National Cancer Center, Goyang, South Korea; ^5^ Department of Pathology, National Cancer Center, Goyang, South Korea

**Keywords:** bladder cancer, recurrence, antibiotics, progression, BCG - Bacille Calmette-Guérin vaccine

## Abstract

**Objective:**

To investigate the association between antibiotic therapy and the efficacy of intravesical BCG therapy in patients with high-risk non-muscle invasive bladder cancer (NMIBC).

**Methods:**

This study involved the retrospective review of medical records of patients who underwent transurethral resection of bladder tumors for high-risk NMIBC followed by intravesical BCG therapy between 2008 and 2017. Patients were categorized as none, short- (2-6 days), and long-course use (≥7 days) based on the duration of antibiotic treatment concurrent with or initiated ≤30 days before BCG therapy. Oncologic outcomes, including recurrence-free survival and progression-free survival, were analyzed.

**Results:**

Of the 276 patients enrolled in the study, 162 (58.7%) had pathologic T1 disease and 206 (80.2%) had high-grade disease. Concurrently with or prior to BCG therapy, 114 patients had (41.3%) received short-course antibiotic therapy, and 96 (34.8%) patients had received long-course antibiotics. The 5-year recurrence-free survival (62.2% vs 26.9%; log rank, p <0.001) and progression-free survival (79.6% vs. 53.3%; log rank, p=0.001) rates were significantly higher in patients who did not receive antibiotic therapy than in those treated with long-course antibiotics. Multivariable analysis revealed that antibiotic treatment for more than 7 days was independently associated with increased risks of recurrence (hazard ratio [HR], 2.45; 95% confidence interval [CI], 1.49-4.05; p < 0.001) and progression (HR, 3.68; 95% CI, 1.65-8.22 p = 0.001).

**Conclusion:**

Long-course antibiotic treatment concurrently with or prior to intravesical BCG adversely influenced disease recurrence and progression outcomes in patients with high-risk NMIBC. Careful use of antibiotics may be required to enhance the efficacy of intravesical BCG therapy. Further mechanistic and prospective studies are warranted.

## Introduction

Non-muscle invasive bladder cancer (NMIBC), which is confined to the mucosa (Ta or carcinoma *in situ*, CIS) or submucosa (T1), accounts for 75% of new cases of bladder cancer (1). The probability of 5-year recurrence of NMIBC is high, ranging from 31% to 78% according to risk stratification (1).

Following transurethral resection of bladder tumor (TUR-BT), intravesical chemotherapy or immunotherapy is the mainstay of NMIBC management (1–3). Intravesical Bacillus Calmette–Guérin (BCG) instillation, which has been used in the treatment of NMIBC for more than 40 years, is superior to intravesical chemotherapy, in terms of disease recurrence and progression, particularly for intermediate- and high-risk NMIBC (3). Nonetheless, up to 50% of patients experience BCG failure (4). In case of BCG-unresponsive high-risk NMIBC, radical cystectomy with pelvic lymph node dissection and urinary diversion is the standard treatment. This procedure is associated with significant morbidity and mortality, and poor health-related quality of life (5). Furthermore, a worldwide BCG shortage contributes to a delay or lack of treatment (6). Thus, it is essential to attempt treatment and optimize the efficacy of intravesical BCG therapy in patients with NMIBC.

The high recurrence rates in NMIBC underscores the importance of predicting disease recurrence and progression (1). The European Organization for Research and Treatment of Cancer (EORTC) Genito-Urinary Cancer Group has published a prediction model for prognosis of NMIBC (7). This model is based on the six essential clinical and pathological parameters: tumor size, grade, T category, presence of concomitant CIS, number of tumors, and prior recurrence rate. Despite the EORTC model being useful to stratify risks of recurrence and progression, there are some limitations in identifying those individuals who will experience a disease recurrence or progression, particularly in patients treated with intravesical BCG (8, 9).

The intestinal microbiome has emerged as a key host determinant of immunity to cancer, and its modulation may influence response to immunotherapy (10). It is known that antibiotic therapy impacts the effectiveness of immunotherapy by modifying the composition of the microbiome composition (11, 12). The implications of urinary microbiome in cancer are unknown. However, there is increasing evidence supporting the importance of the urinary microbiome in the progression of urothelial cancers (13, 14). Owing to its local immunoinflammatory response, the urinary microbiome may influence the outcomes of intravesical BCG therapy (13). We hypothesized that antibiotic therapy concurrent or prior to BCG therapy may influence the efficacy of intravesical BCG, by modulating urinary microbiome composition. The aim of this study was to investigate the association between antibiotic therapy and the efficacy of intravesical BCG therapy in patients with high-risk NMIBC.

## Methods

The medical records of 321 patients subjected to TUR-BT for high-risk NMIBC and who then received intravesical BCG therapy at the National Cancer Center Korea between 2008 and 2017 were retrospectively reviewed. Patients with the following findings were excluded: muscle invasive bladder cancer (n = 4); incomplete (<5 weeks) induction BCG therapy (n = 10); incomplete TUR-BT without second resection (n = 1); or inadequate clinical data (n = 27). Baseline demographic and clinicopathological characteristics, treatment-related variables, antibiotic therapy, disease recurrence, progression, and survival outcomes were evaluated. The study protocol was approved by the institutional review board of National Cancer Center (no. 2019-0293).

All patients underwent TUR-BT prior to intravesical induction BCG therapy. Following TUR-BT, cystourethroscopic examination, urine cytology, and urinalysis were performed every 3 months for the first 2-3 years, every 6 months for the third and fourth years, and annually thereafter. Pathological characteristics included the tumor stage, grade, size, and number of lesions, and variant histology. Treatment-related evaluation included number of previous TUR-BT, prior recurrence rate, abdominopelvic computed tomography, urine cytology, urinalysis, second resection procedure, and administration of maintenance intravesical BCG. Maintenance BCG was given once per week for three weeks at 3, 6, and 12 months after the initial BCG treatment. The duration, timing, class, and indication of antibiotic therapy were analyzed. Patients were classified in to three antibiotic groups based on the duration of antibiotic therapy as: concurrent or ≤ 30 days prior to BCG therapy: no antibiotics (none); 2-6 days (short-course use); and > 7 days (long-course use). All patients received prophylactic antibiotics (cephalosporin or fluoroquinolone) immediately before TUR-BT. Therefore, antibiotic therapy was defined when antibiotics were prescribed for two or more days. Decisions regarding post-TUR-BT management and need for antibiotics were made by urologists.

Oncologic outcomes, including recurrence-free survival (RFS) and progression-free survival (PFS) were analyzed. Cystourethroscopic examination, urine cytology, and computed tomography were performed to assess disease recurrence following TUR-BT. Recurrence was defined as histologically confirmed urothelial cancer on tumor tissue. Progression was recorded when patients showed progression to pathologic muscle-invasive urothelial cancer or extravesical disease.

Patient characteristics were expressed as median or mean for continuous variables and as frequency for categorical variables. Comparison of the groups according to duration of antibiotic therapy was performed using the t-test, analysis of variance (ANOVA), and chi-square test. Survival outcomes were estimated using the Kaplan-Meier method, and the log-rank test was used to test for differences in the survival curves. Cox proportional hazard models were used to identify significant predictors of recurrence-free survival and progression-free survival. A p-value < 0.05 was indicated statistical significance. All statistical analyses were performed using SAS 9.4 (SAS Institute Inc., Cary, NC, USA) and R 3.5.2 version (R Foundation for Statistical Computing, Vienna, Austria).

## Results

A total of 276 patients with NMIBC were included for analysis. Patient characteristics are summarized in **Table 1**. The median follow-up duration was 55 months. Of the 276 patients, 162 (58.7%) had pathologic T1 disease and 225 (81.5%) had high-grade disease. Age, sex, T stage, tumor grade, lymphovascular invasion, number of previous TUR-BT, second resections, and administration of maintenance intravesical BCG did not differ significantly among the various antibiotic groups.

**Table 1:** Baseline characteristics of patients.

	Overall	Antibiotic therapy			P-value
		No	Short-course	Long-course	
Total, n	276	66 (23.9)	114 (41.3)	96 (34.8)	
Age (median, yr.)	68	67	67	69	0.9
Gender					0.7
Male	220 (79.7)	54 (81.8)	88 (77.2)	78 (81.3)	
Female	56 (20.3)	12 (18.2)	26 (22.8)	18 (18.8)	
ECOG performance status ≥1	23 (8.7)	4 (6.1)	11 (10.1)	8 (9.1)	0.7
Pathologic T stage					0.7
Ta	114 (41.3)	27 (40.9)	50 (43.9)	37 (38.5)	
T1	162 (58.7)	39 (59.1)	64 (56.1)	59 (61.5)	
Tumor grade					0.4
Low	51 (18.5)	13 (19.7)	17 (14.9)	21 (21.9)	
High	225 (81.5)	53 (80.3)	97 (85.1)	75 (78.1)	
Tumor size					0.3
<3 cm	213 (77.2)	52 (78.8)	83 (72.8)	78 (81.3)	
≥3 cm	63 (22.8)	14 (21.2)	31 (27.2)	18 (18.8)	
Number of tumors					0.9
Single	116 (42.0)	24 (36.4)	50 (43.9)	42 (43.8)	
2 to 7	85 (30.8)	21 (31.8)	35 (30.7)	29 (30.2)	
≥ 8	75 (27.2)	21 (31.8)	29 (25.4)	25 (26.0)	
Concurrent carcinoma in situ	69 (25.0)	11 (16.7)	34 (29.8)	24 (25.0)	0.2
Lymphovascular invasion	20 (7.4)	2 (3.0)	13 (11.4)	5 (5.2)	0.07
Histologic variants	14 (5.1)	1 (1.5)	7 (6.1)	6 (6.3)	0.3
No. of previous TUR-BT (median)	2	2	1	2	0.6
Prior recurrence rate					0.3
Primary	137 (49.6)	32 (48.5)	56 (49.1)	49 (51.0)	
≤1 recurrence/yr.	70 (25.4)	21 (31.8)	31 (27.2)	18 (18.8)	
>1 recurrence/yr.	69 (25.0)	13 (19.7)	27 (23.7)	29 (30.2)	
Repeated TUR-BT	158 (57.2)	38 (57.6)	70 (61.4)	50 (52.1)	0.4
BCG maintenance	124 (44.9)	31 (47.0)	55 (48.2)	38 (39.6)	0.4
Urinalysis before BCG therapy					
RBC ≥5 per HPF	200 (72.5)	48 (72.7)	84 (73.7)	68 (70.8)	0.9
WBC ≥5 per HPF	148 (53.6)	39 (59.1)	58 (50.9)	51 (53.1)	0.6
Nitrite positive	4 (1.4)	0	1 (0.9)	3 (3.1)	0.6
Urine culture before BCG therapy					0.9
Negative	259 (93.8)	63 (95.5)	107 (93.9)	89 (92.7)	
*E. coli*	8 (2.9)	2 (3.0)	3 (2.6)	3 (3.1)	
Others	9 (3.3)	1 (1.5)	4 (3.5)	4 (4.2)	
Class of antibiotics					
Quinolone		–	84 (73.7)	82 (85.4)	
Cephalosporin		–	30 (26.3)	14 (14.6)	
Timing of antibiotic treatment					
Prior only		–	75 (65.8)	38 (39.6)	
Concurrent only		–	14 (12.3)	17 (17.7)	
Prior+concurrent		–	24 (21.1)	41 (42.7)	

ECOG, Eastern Cooperative Oncology Group; RBC; red blood cells; WBC, white blood cells; TUR-BT, transurethral resection of bladder tumor.

Values are presented as number and percentages.

Out of 276 patients, 114 (41.3%) received short-course antibiotic therapy and 96 (34.8%) patients received long-course antibiotics. Prior to BCG therapy, 17 (6.2%) patients showed a positive result in urine culture. Of a total of 17 patients having positive urine culture prior to surgery, fluoroquinolone-resistant pathogens were found in 15 patients (88.2%) and ESBL-producing pathogens in 10 (58.8%). The most common reasons for antibiotic therapy were dysuria (43.1%) and prophylaxis (46.9%). Among the patients who received antibiotic therapy, quinolones and cephalosporins were prescribed in 166 (ciprofloxacin: 122; levofloxacin: 44) patients and 44 (cefaclor: 23; cefpodoxime: 14; cefixime: 7) patients, respectively.

**Figure 1:**
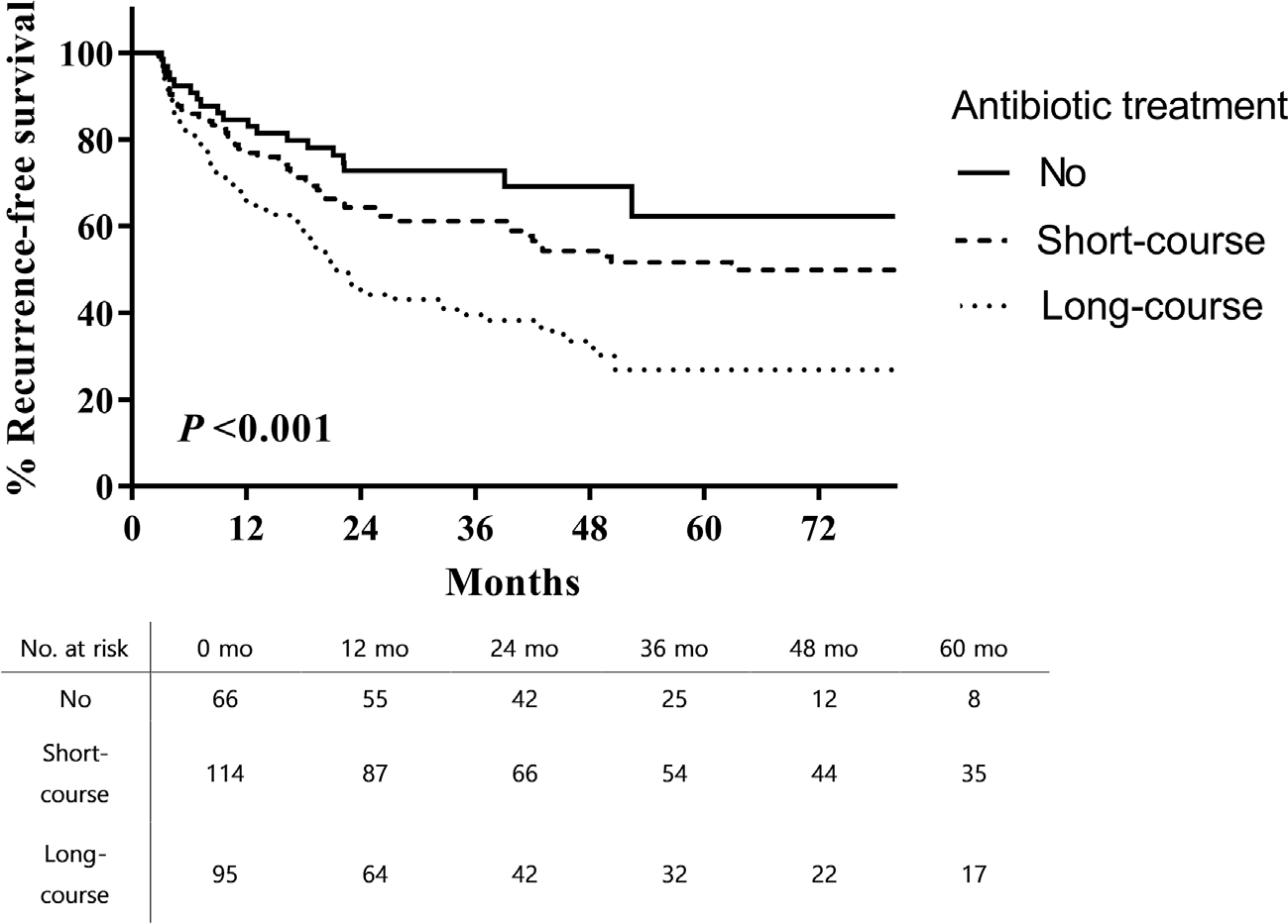
Recurrence-free survival after intravesical BCG therapy in patients with high-risk non-muscle invasive bladder cancer.

**Figure 2:**
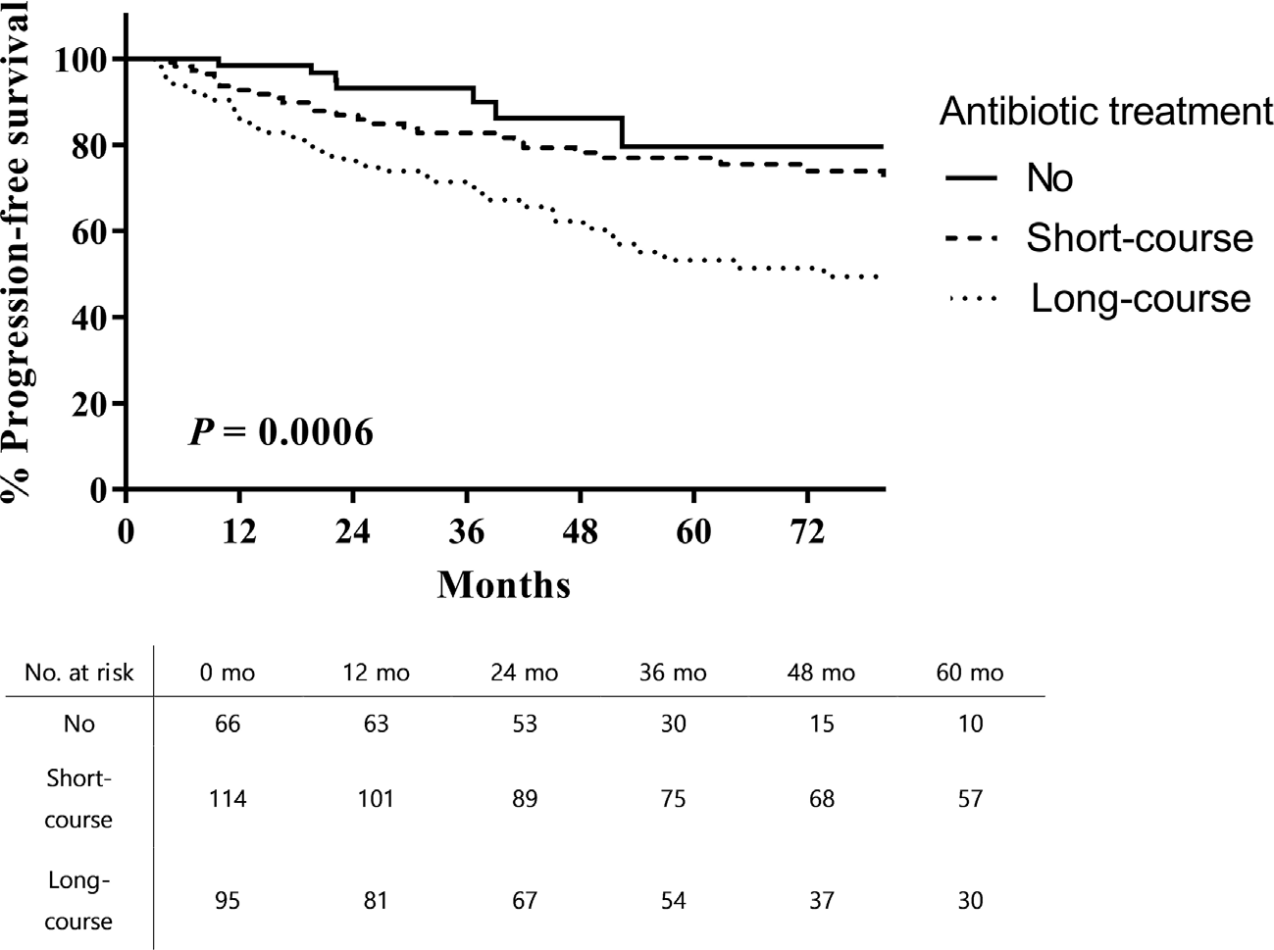
Progression-free survival after intravesical BCG therapy in patients with high-risk non-muscle invasive bladder cancer.

The 5-year RFS from the time of TUR-BT was significantly higher in patients who did not receive antibiotic therapy than in those who received long-course antibiotic therapy (62.2% vs. 26.9%; log rank, p < 0.001; **Figure 1**). PFS was also higher in patients who did not receive antibiotic therapy than in those who received long-course antibiotic therapy (79.6% vs. 53.3%; log rank, p = 0.0006; **Figure 2**).

Multivariable analysis (**Supplementary Table 1**) showed that long-course antibiotic treatment was independently associated with an increased risk of recurrence (hazard ratio [HR], 2.45; 95% confidence interval [CI], 1.49-4.05; p < 0.001) and progression (HR, 3.68; 95% CI, 1.65-8.22 p = 0.001); the other factors included tumor grade, concurrent CIS, and maintenance BCG therapy. Interaction tests showed that the relationship between antibiotic treatment and outcomes was unrelated to antibiotic class or timing (all p > 0.05).

## Discussion

In the present study, we found that long-course antibiotic therapy was independently associated with recurrence and progression following intravesical BCG therapy in patients with high risk NMIBC. Because of the high recurrence and progression probability after TUR-BT, intravesical therapy plays a major role in managing NMIBC (1–3). Intravesical BCG therapy is superior to intravesical chemotherapy (mitomycin or gemcitabine) in reducing NMIBC recurrence, particularly in intermediate- and high-risk disease (4, 15). In addition, owing to the worldwide shortage of BCG, there is an urgent need to predict and maximize the efficacy of intravesical BCG therapy. Despite numerous efforts to predict and potentiate the effects of intravesical BCG, only a few generally accepted biomarkers or administration methods are available in clinical practice (14, 16). Maintenance BCG therapy is required to reduce both recurrence and progression. However, there is no consensus on the optimal duration of maintenance BCG therapy (3, 15). Consistent with the established literature, our study found that maintenance BCG therapy was associated with recurrence-free and progression-free survival benefits.

Antibiotics are often prescribed to manage or prevent intravesical BCG-related side effects (10). Common adverse events of BCG instillation include lower urinary tract symptoms (27-95%), hematuria (1-40%), urinary tract infection (5%), and low-grade fever (30%) (10). Broad-spectrum antibiotic treatments are indicated for BCG sepsis, a rare but life-threatening complication. In the present study, antibiotics were prescribed either for dysuria or as prophylaxis in most cases. However, antibiotics were somewhat overused in our institution, considering the low positivity reported by urine culture (6.7%), which may be related with national practice patterns and policy (17, 18). In this study, long-course (≥7 days) antibiotic therapy was found to be independently associated with poorer outcomes. Recurrence-free survival and progression-free survival rates were lower in patients who received short-course antibiotic therapy than in those who did not receive antibiotics; however, short-course therapy was not an independent predictor of outcome. Antibiotic class and administration timing did not appear to have any influence on these outcomes.

Several studies have investigated the relationship between antibiotic use and the efficacy of intravesical BCG. Van Der Meijden et al. reported the possible inhibition of antitumor efficacy by eradication of BCG organisms and suggested that intravesical therapy should not be accompanied by antibiotics in the absence of cystitis (19), corresponding to our results. In contrast, one large randomized trial by the EORTC showed that concurrent use of isoniazid showed no difference in efficacy with combination therapy (20). Regarding antibiotic treatment that did not target tuberculosis, Colombel et al. reported that prophylactic ofloxacin was not associated with recurrence and progression at the one year post-surgery follow-up (21). Damiano et al. also reported that short-course prulifloxacin was not associated with recurrence rates at 6 months (22). However, both studies had a relatively shorter follow-up duration and analyzed the effects of short-course antibiotic therapy, unlike our study, and may have contributed to the difference in results. Our results suggest that long-course antibiotic therapy may reduce the efficacy of intravesical BCG after TUR-BT; thus, avoiding unnecessary antibiotic therapy may be helpful in the management of high risk NMIBC.

Microbiota are a diverse consortium of commensal, symbiotic, and pathogenic microorganisms. The role of gut microbiota in the development of various malignancies is well established (10, 13). In addition, the association between gut microbiota modulation and treatment response in cancer patients has been extensively studied, especially with the use of immunotherapy (10). Recent studies have disproved the dogma that urine is sterile (13, 14, 23). While most urinary microbiome-related studies concern urinary tract infections, there are some preliminary data demonstrating an association between the urinary microbiome and urothelial carcinoma (24, 25).

One possible mechanism of action of intravesical BCG therapy is by bladder microbiome manipulation (13, 26). BCG is a live attenuated form of *Mycobacterium bovis*, which attaches to the urothelial fibronectin, resulting in direct tumor and immune response (15). In this process, local microorganisms may potentially interact with BCG, influencing immunity to bladder cancer (23). It is well known that antibiotic therapy modulates intestinal microbiota (27). Accordingly, several studies have reported that antibiotic treatment adversely influenced response to immune checkpoint inhibitors through the modulation of the gut microbiota (11, 12). Based on the above evidence, we hypothesized that antibiotic therapy concomitant or prior to BCG therapy may influence therapeutic efficacy, because of the potential relationship between antibiotic use, the urinary microbiome, and the mechanism action of intravesical BCG. Although we cannot elucidate underlying mechanisms involved to fully explain our results, it is possible that the efficacy of BCG is related to the composition of the urinary microbiome, which, in turn, is influenced by long-course antibiotic treatment.

This study had several limitations. First, the retrospective nature of the study was itself susceptible to inherent bias. Inherent limitations may have included selection bias for antibiotic treatment and the lack of standardized protocols for TUR-BT, second resection, and maintenance BCG therapy. Therefore, only limited conclusions can be drawn from this study, which require further prospective validation for confirmation. Second, the definition of prior antibiotic therapy and long-course antibiotic therapy may be inappropriate. We used a 30-day cut-off point from antibiotic exposure to BCG therapy based on the results of a longitudinal study indicating that compositional changes in the gut microflora recovers about one month after antibiotic dosing (28). However, there are few reliable data available regarding the relationship between antibiotic therapy and urinary microbiome changes (29, 30). Third, this was not a mechanistic study. The hypothesis that the urinary microbiome responds to intravesical BCG, and its possible modulation by antibiotics should be explored in future studies. Despite these limitations, we believe that this study provides a valuable contribution to the literature as the first study to demonstrate the impact of long-course antibiotic therapy on the outcomes of intravesical BCG in patients with high-risk NMIBC. We think that prior or concurrent antibiotic treatment, particularly long-term use, should be avoided in patients who underwent intravesical BCG without symptomatic bacteriuria. Our study results also suggest the need for further investigation of the potential relationship between the urinary microbiome and bladder cancer.

In summary, long-course antibiotic treatment concomitant or prior to intravesical BCG therapy adversely influences disease recurrence and progression outcomes in patients with high risk NMIBC. Careful prescription of antibiotics may help to enhance the efficacy of intravesical BCG therapy. Further mechanistic and randomized studies are warranted to fully comprehend the influence of antibiotic therapy in patients with bladder cancer.

## Data Availability Statement

The raw data supporting the conclusions of this article will be made available by the authors, without undue reservation.

## Ethics Statement

The studies involving human participants were reviewed and approved by National Cancer Center. Written informed consent for participation was not required for this study in accordance with the national legislation and the institutional requirements.

## Author Contributions

SP designed the study, performed the data analysis, and drafted the manuscript. S-YK performed statistical analysis. SK, JJ, WP, JC, and KL participated in the data acquisition. HS supervised the project. All authors discussed the results and commented on the manuscript. All authors contributed to the article and approved the submitted version.

## Funding

This work was supported by a research grant (No. 1810242) from the National Cancer Center, Republic of Korea.

## Conflict of Interest

The authors declare that the research was conducted in the absence of any commercial or financial relationships that could be construed as a potential conflict of interest.
