# Supplementary material for: Association Between Antibiotic Treatment and the Efficacy of Intravesical BCG Therapy in Patients With High-Risk Non-Muscle Invasive Bladder Cancer
Source: Front Oncol. 2021 Apr 2;11:570077. doi: 10.3389/fonc.2021.570077 (PMC8051584; doi:10.3389/fonc.2021.570077)
Supplement: Supplementary file 1 [file Table_1.docx]

Supplementary Table 1A. Multivariable analysis for predicting risk of disease recurrence.

|  | Univariate |  |  | Multivariate |  |  |
| --- | --- | --- | --- | --- | --- | --- |
|  | Hazard ratio | 95% CI | P-value | Hazard ratio | 95% CI | P-value |
| Age | 1.02 | 1.00-1.04 | 0.018 | 1.02 | 1.00-1.04 | 0.019 |
| Pathologic T stage (T1 vs. Ta) | 1.17 | 0.84-1.63 | 0.4 |  |  |  |
| Tumor grade (high vs. low) | 1.21 | 0.81-1.79 | 0.3 |  |  |  |
| Concurrent CIS | 1.36 | 0.95-1.95 | 0.090 | 1.48 | 1.02-2.13 | 0.037 |
| Tumor size (≥3 vs. <3 cm) | 1.34 | 0.91-2.10 | 0.130 |  |  |  |
| Number of tumors |  |  |  |  |  |  |
| Single | Reference |  |  |  |  |  |
| 2 to 7 | 0.74 | 0.49-1.11 | 0.149 |  |  |  |
| ≥ 8 | 0.98 | 0.66-1.45 | 0.9 |  |  |  |
| Prior recurrence rate |  |  |  |  |  |  |
| Primary | Reference |  |  |  |  |  |
| ≤1 recurrence / yr | 1.42 | 0.95-2.11 | 0.085 |  |  |  |
| >1 recurrence / yr | 1.20 | 0.80-1.80 | 0.4 |  |  |  |
| Repeated TURB | 1.07 | 0.76-1.49 | 0.7 |  |  |  |
| BCG maintenance | 0.50 | 0.35-0.71 | <0.001 | 0.49 | 0.34-0.69 | <0.001 |
| Antibiotic treatment |  |  |  |  |  |  |
| None | Reference |  |  | Reference |  |  |
| Short-course (2-6 days) | 1.44 | 0.86-2.41 | 0.170 | 1.33 | 0.79-2.24 | 0.287 |
| Long-course (≥7 days) | 2.62 | 1.60-4.32 | <0.001 | 2.45 | 1.49-4.05 | <0.001 |

All factors with p<0.1 in univariate analyses were included in the Cox multivariate analysis. CI; confidence interval.

Supplementary Table 1B. Multivariable analysis for predicting risk of disease progression.

|  | Univariate |  |  | Multivariate |  |  |
| --- | --- | --- | --- | --- | --- | --- |
|  | Hazard ratio | 95% CI | P-value | Hazard ratio | 95% CI | P-value |
| Age | 1.03 | 1.01-1.05 | 0.007 | 1.03 | 1.00-1.05 | 0.015 |
| Pathologic T stage (T1 vs. Ta) | 1.14 | 0.73-1.79 | 0.6 |  |  |  |
| Tumor grade (high vs. low) | 2.00 | 1.03-3.90 | 0.041 | 2.12 | 1.08-4.19 | 0.030 |
| Concurrent CIS | 1.62 | 1.02-2.56 | 0.040 | 1.55 | 0.97-2.47 | 0.068 |
| Tumor size (≥3 vs. <3 cm) | 1.32 | 0.74-2.36 | 0.3 |  |  |  |
| Number of tumors |  |  |  |  |  |  |
| Single | Reference |  |  |  |  |  |
| 2 to 7 | 0.99 | 0.60-1.65 | 0.9 |  |  |  |
| ≥ 8 | 0.72 | 0.41-1.27 | 0.3 |  |  |  |
| Prior recurrence rate |  |  |  |  |  |  |
| Primary | Reference |  |  |  |  |  |
| ≤1 recurrence / yr | 1.14 | 0.67-1.96 | 0.6 |  |  |  |
| >1 recurrence / yr | 1.15 | 0.68-1.96 | 0.6 |  |  |  |
| Repeated TURB | 1.23 | 0.78-1.93 | 0.4 |  |  |  |
| BCG maintenance | 0.53 | 0.33-0.85 | 0.009 | 1.72 | 0.75-3.96 | 0.002 |
| Antibiotic treatment |  |  |  |  |  |  |
| None | Reference |  |  | Reference |  |  |
| Short-course (2-6 days) | 1.86 | 0.81-4.27 | 0.141 | 1.72 | 0.75-3.96 | 0.2 |
| Long-course (≥7 days) | 3.66 | 1.64-8.17 | 0.002 | 3.68 | 1.65-8.22 | 0.001 |

All factors with p<0.1 in univariate analyses were included in the Cox multivariate analysis. CI; confidence interval.
